# Diet-induced obesity leads to behavioral indicators of pain preceding structural joint damage in wild-type mice

**DOI:** 10.1186/s13075-021-02463-5

**Published:** 2021-03-22

**Authors:** Geoffrey J. Kerr, Bethia To, Ian White, Magali Millecamps, Frank Beier, Matthew W. Grol, Laura S. Stone, Cheryle A. Séguin

**Affiliations:** 1 Department of Physiology & Pharmacology, Schulich School of Medicine & Dentistry, Bone and Joint Institute, The University of Western Ontario, London, Ontario N6A 5C1 Canada; 2 Alan Edwards Centre for Research on Pain, Faculty of Dentistry, McGill University, Montreal Quebec, Canada; 3 Department of Anesthesiology, Faculty of Medicine, University of Minnesota, Minneapolis Minnesota, USA

**Keywords:** Obesity, High-fat diet, Western diet, Behavioral measures of pain, Intervertebral disc degeneration, Osteoarthritis

## Abstract

**Introduction:**

Obesity is one of the largest modifiable risk factors for the development of musculoskeletal diseases, including intervertebral disc (IVD) degeneration and back pain. Despite the clinical association, no studies have directly assessed whether diet-induced obesity accelerates IVD degeneration, back pain, or investigated the biological mediators underlying this association. In this study, we examine the effects of chronic consumption of a high-fat or high-fat/high-sugar (western) diet on the IVD, knee joint, and pain-associated outcomes.

**Methods:**

Male C57BL/6N mice were randomized into one of three diet groups (chow control; high-fat; high-fat, high-sugar western diet) at 10 weeks of age and remained on the diet for 12, 24, or 40 weeks. At endpoint, animals were assessed for behavioral indicators of pain, joint tissues were collected for histological and molecular analysis, serum was collected to assess for markers of systemic inflammation, and IBA-1, GFAP, and CGRP were measured in spinal cords by immunohistochemistry.

**Results:**

Animals fed obesogenic (high-fat or western) diets showed behavioral indicators of pain beginning at 12 weeks and persisting up to 40 weeks of diet consumption. Histological indicators of moderate joint degeneration were detected in the IVD and knee following 40 weeks on the experimental diets. Mice fed the obesogenic diets showed synovitis, increased intradiscal expression of inflammatory cytokines and circulating levels of MCP-1 compared to control. Linear regression modeling demonstrated that age and diet were both significant predictors of most pain-related behavioral outcomes, but not histopathological joint degeneration. Synovitis was associated with alterations in spontaneous activity.

**Conclusion:**

Diet-induced obesity accelerates IVD degeneration and knee OA in mice; however, pain-related behaviors precede and are independent of histopathological structural damage. These findings contribute to understanding the source of obesity-related back pain and the contribution of structural IVD degeneration.

## Background

Obesity—traditionally defined as a body mass index over 30—is a worldwide epidemic. Obesity substantially increases the risk of developing metabolic, cardiovascular, neurological, and musculoskeletal diseases [1], and with the prevalence nearly tripling over the last 30 years [1], it poses a large public health concern. Obesity decreases both life expectancy [2] and quality of life and is associated with increased disability, mental illness, and unemployment [1, 3]. A significant contributor to obesity-induced disability is low back pain (LBP) [4, 5], which is the single most common cause of long-term pain and disability worldwide [6]. Despite efforts to improve the clinical management of LBP, treatments are largely limited to symptomatic relief, often without treating the underlying cause of the pain [7]. This is largely due to an incomplete understanding of the tissues and pathways involved in the initiation and progression of LBP. While several tissues appear to be involved in LBP, including the paraspinal muscles, ligaments, and facet joints [8–10], degeneration of the fibrocartilaginous intervertebral disc (IVD) is believed to be the major contributor to pain in an approximately 40% of cases [8].

Despite the clinical associations between LBP, IVD degeneration, and obesity, the underlying mechanisms and biological pathways responsible remain unknown. One contributing factor appears to be increased mechanical loading. Excess weight alters the mechanical load experienced by the IVD [11], a known regulator of IVD cellular function [12, 13]. Increased body weight is associated with indices of lumbar disc degeneration including disc space narrowing and decreased lumbar disc signal intensity detected by MRI [14–16]. In articular cartilage, excess weight and altered mechanical loading has also been suggested to contribute to osteoarthritis (OA) [16], a degenerative musculoskeletal disease with many similarities to IVD degeneration [17]. Of note, increased mechanical load alone does not account for the association between obesity and OA, as obese individuals also present more frequently with OA in non-weight bearing joints, such as the hand [18].

In addition to increased mechanical load, metabolic abnormalities associated with obesity impact musculoskeletal health [19, 20]. Obesity is associated with chronic metabolic disorders including hypertension, diabetes mellitus, and dyslipidemia, collectively known as metabolic syndrome [21]. In the context of OA, it is postulated that each component of metabolic syndrome may independently contribute to disease progression, as comprehensively reviewed by Zhuo et al. [22] Specifically, alterations in the release of systemic factors (inflammatory cytokines, adipokines), nutrient exchange, advanced glycation end-products (AGEs) levels, and glucose/lipid metabolism are believed to be major contributors to OA progression [19, 22]. Studies from multiple groups have demonstrated using mouse models that obesity induced by a high-fat diet accelerates the progression of both age- and surgically induced knee OA [23–27], accompanied by behavioral indicators of pain [23]. Aside from its role in energy storage, adipose tissue is also a major endocrine organ and has been shown to secrete hormones termed adipokines (e.g., leptin, adiponectin, visfatin, resistin) and inflammatory cytokines (e.g., TNF-α, IL-6, TGF-β) [28]. Studies investigating the role of adipokines have highlighted their importance in obesity-associated pathologies. For example, leptin-deficient mice become obese yet they do not develop knee OA, suggesting leptin may play a key role in obesity-induced OA [29]. In the IVD, exposure of nucleus pulposus (NP) cells to adipokines, such as leptin and resistin, promotes catabolic metabolism associated with increased expression of matrix remodeling enzymes such as MMP and ADAMTS genes [30, 31]. Adipokines also appear to play a role systemically as modulators of pain sensitivity [32]. In addition to back pain, obese individuals are more likely to develop chronic pain conditions such as fibromyalgia, headaches, and abdominal pain [33]. While the underlying mechanisms linking obesity and chronic pain remains unknown, it has been suggested that systemic immune and endocrine alterations play a role in the altered pain response [34]. This systemic modulation of pain may contribute to LBP in addition to structural alterations and local inflammation within the IVD itself.

While there is extensive clinical evidence supporting the association between obesity, LBP, and IVD degeneration [35, 36], no studies have directly assessed whether diet-induced obesity accelerates IVD degeneration, back pain, or investigated biological mediators underlying this association. The current study was designed to investigate whether chronic consumption of a high-fat or high-fat, high-sugar western diet alters the progression of age-related IVD degeneration or back pain using the mouse as a model.

## Materials and methods

### Mice and diets

Wild-type, male, C57BL/6N (Charles River: Wilmington, MA, USA) mice were fed standard chow (Envigo 2018) after weaning and randomized at 10 weeks of age into one of three diet groups (*n* = 9–16 mice/group; Supplementary Table 1) based on previous reports of obesity and metabolic derangement in mice [26, 37]: high-fat diet (60% kcal fat, 21% kcal carbohydrate; Envigo TD.06414), western diet (45% kcal fat, 41% kcal carbohydrate; Envigo TD.10885), or standard chow (18% kcal fat, 58% kcal carbohydrate). Mice remained on the experimental diets until sacrifice at 5, 8, or 11.5 months-of-age (12, 24, 40 weeks on diet, respectively). Mice were housed in standard cages and maintained on a 12 h light/dark cycle, with food and water consumed ad libitum; food consumption and body weight were measured weekly. All aspects of this study were conducted in accordance with the policies and guidelines set forth by the Canadian Council on Animal Care and were approved by the Animal Use Subcommittee of the University of Western Ontario (protocol 2017–154).

### Characterization of pain-associated behaviors

Behavioral analysis was conducted on mice following 12, 24, or 40 weeks on experimental or control diets. Behavioral studies were preceded by a 2-week habituation to the neurobehavioral testing facility, and mice were habituated to all tests 1 week prior to data collection. On data collection days, animals were habituated to the testing room for 1 h before test start. To avoid confounding variables associated with the diurnal cycle, all behavioral assessments were conducted between 8 and 11 AM.

#### Stretch-induced axial discomfort

Stretch-induced axial discomfort was measured using the tail suspension test and grip force during axial stretch, as described previously [38–40]. For the tail suspension test, spontaneous reaction to gravity-induced stretch was assessed in mice suspended by the base of their tails for 180 s. Two observers blinded to the experimental groups independently scored video recordings for the duration of time spent by mice in immobility, full extension, rearing, or self-supporting using ANY-maze software (Stoelting Co.: Wood Dale, IL). Voluntary activity was quantified for 5 min immediately before (pre) and after (post) tail suspension using open-field activity monitors (AccuScan Instruments, Omnitech Electronic: Columbus, OH), to quantify movement-evoked discomfort. The difference in total distance between the two open-field sessions (post-pre) was calculated for each mouse.

For the grip force assay, mice were positioned to grab onto a metal bar attached to a grip force meter (Stoelting Co.: Wood Dale, IL) and then gently pulled back by their tails to exert axial stretch. Tolerance was assessed by measuring the grip strength, in grams, for each mouse at the point of release averaged over 3 trials.

#### Hind limb sensitivity to mechanical and cold stimuli

Mechanical sensitivity was measured through application of calibrated Von Frey filaments (Stoelting Co.: Wood Dale, IL) to the plantar surface of the hind paw for 3 s or withdrawal. Fifty-percent withdrawal threshold was calculated using the Chaplan up-down method [41]. The stimulus intensity ranged from 0.07 to 6.0 g, beginning with a stimulus intensity of 1.4 g. Cold sensitivity was assessed by measuring the total time spent by mice in behavior evoked by evaporative cooling of acetone (flicking, stamping, or licking of ventral surface of the paw) during the first 40 s following application of 50 μL acetone to the ventral surface of the hind paw. The test was carried out twice for each paw, with at least 5 min recovery between each test. Times were then averaged between paws.

#### Spontaneous activity

Voluntary locomotor activity was assessed using open field activity monitors (AccuScan Instruments, Omnitech Electronic: Columbus, OH). Mice were placed into individual boxes and their activity was monitored over 2 h. This was repeated for 3 consecutive days and values were averaged for each mouse.

### Micro-computed tomography (micro-CT)

Forty-eight hours before sacrifice, μCT imaging was performed using a cone-beam imaging system (eXplore SpeCZT scanner, GE Healthcare Biosciences: London, CAN). For imaging, mice were anesthetized using 2–3% inhaled isoflurane (CA2L9100, Baxter: Mississauga, CAN) infused with oxygen at a flow rate of 1.0 mL/min. To maintain sedation, a nose cone apparatus was used to administer 1.75% inhaled isoflurane for 20 min while scanning was performed. During a single 5 min rotation of the gantry, 900 X-ray projections were acquired (peak voltage of 90 kVp, peak tube current of 40 mA, and integration time of 16 ms). A calibrating phantom composed of air, water, and cortical bone-mimicking epoxy (SB3; Gammex, Middleton WI, USA) was included in each scan. Data were reconstructed into 3D volumes with an isotropic voxel spacing of 50 μm and scaled into Hounsfield units (HU). Using MicroView software (GE Healthcare Biosciences) three signal-intensity thresholds (− 200, − 30, and 190 HU) were used to classify each voxel as adipose, lean, or skeletal tissue, respectively. Custom software was used to calculate tissue masses from assumed densities of 0.95 (adipose), 1.05 (lean), and 1.92 (skeletal) g/cm^3^, as previously reported [42].

### Histological analysis

Intact lumbar spine segments (L1-S1) and knees were isolated, fixed, decalcified, and paraffin embedded, as previously described [43]. Spines were sectioned sagittally, and knees were sectioned coronally at a thickness of 5 μm using a microtome (Leica Microsystems: Wetzlar, DEU). Lumbar spines were stained using a 0.1% Safranin-O/0.05% Fast Green. Knees were stained with 0.04% Toluidine Blue. Sections were imaged on a Leica DM1000 microscope, with Leica Application Suite (Leica Microsystems: Wetzlar, DEU).

To evaluate IVD degeneration, spine sections were scored by two blinded observers using the modified Boos system [44]. Knee joint health was assessed using the murine Osteoarthritis Research Society International (OARSI) histopathological scale [45]. Articular surfaces of the medial femoral condyle (MFC), medial tibial plateau (MTP), lateral femoral condyle (LFC), and lateral tibial plateau (LTP) were scored by two blinded observers and averaged. For each knee joint surface, scores from 10 serial sections spanning 500 μm of the joint were summed to represent OARSI score for each quadrant. Total scores from each of the four quadrants were then added together to generate whole joint OARSI score. To assess synovitis, a 3-point histopathological scale [46] was used to evaluate synovial hyperplasia and inflammatory infiltration by a blinded observer. For each mouse, scores corresponding to both the medial and lateral synovium were summed across 7 serial sections. Thickness of articular cartilage of the medial tibial plateau (MTP) and LTP was quantified using the OsteoMeasure7 Program (v.4.2.0.1, OsteoMetrics Inc., Decatur, GA, USA). Articular cartilage thickness within each quadrant was averaged using three serial sections spanning 150 μM of the weight-bearing region of the knee. Using the same histomorphometry system, trabecular bone area was calculated by measuring the total surface area of the bone between the articular cartilage and growth plate and subtracting the area of bone marrow. Measurements were taken for both medial and lateral compartments of the joint and averaged from 3 serial sections.

### Gene expression analysis

Intact thoracic IVDs (inclusive of NP, AF, and CEP) (4–5 per mouse; 5–8 mice per diet/per timepoint) were isolated by microdissection, placed in TRIzol reagent (Thermo Fisher Canada: Mississauga, ON, CAN) and homogenized using a PRO250 tissue homogenizer (PRO Scientific: Oxford, CT, USA). RNA was extracted according to manufacturer’s instructions, quantified using a NanoDrop 2000 spectrophotometer (Thermo Fisher Canada: Mississauga, ON, CAN), and 0.5 μg was reverse transcribed into complementary DNA (cDNA) (iScript; Bio-Rad Laboratories (Canada): Mississauga, ON, CAN). Gene expression was assessed by real-time PCR using a Bio-Rad CFX384 instrument. PCR analyses were run in triplicate using 120 ng of cDNA per reaction and 310 nM forward and reverse primers with 2x SsoFast EvaGreen Supermix (Bio-Rad Laboratories (Canada): Mississauga, ON, CAN) using optimized PCR parameters and primers (Supplementary Table 2). Primers were designed and validated to have efficiency values between 90 and 120%. Transcript levels were calculated relative to a 6-point standard curve made from pooled cDNA generated from murine IVD explants treated with lipopolysaccharide for 4 days (50 mg/mL; Thermo Fisher Canada: Mississauga, ON, CAN).

### Immunohistochemistry

The intact spinal cord was removed, separated into the upper (L1-L2) and lower (L3-L6) segments of the lumbar enlargement and fixed in 4% paraformaldehyde (PFA) for 24 h at 4 °C. Tissues were cryoprotected for 4 days in 10% sucrose and embedded in optimal cutting temperature compound (Tissue-Tek O.C.T; Sakura Finetek US: Torrance, CA, USA) and stored at − 20 °C. Tissues were sectioned on a cryostat (Leica Microsystems: Wetzlar, DEU) in the transverse plane at a thickness of 14 μm, thaw mounted onto gelatin-coated slides, and stored at − 80 °C.

Slides were brought to room temperature, washed twice in PBS and blocked using 5% donkey serum, 0.1% Triton X-100 in PBS for 2 h at room temperature. Sections were incubated overnight in a humidified chamber at 4 °C in 5% donkey serum in PBS (with 0.1% Triton-X) containing primary antibodies directed against glial fibrillary acidic protein (GFAP) (1:500; G3893, Sigma-Aldrich: St. Louis, MO, USA), ionized calcium-binding adaptor molecule 1 (IBA-1) (1:1000; AB-10341, Abcam: Cambridge, UK), or calcitonin gene-related peptide (CGRP) (1:750; BML-CA1137, Enzo Biochem: New York, NY, USA). Slides were rinsed 3 × 10 min in PBS-T (PBS + 0.01% Triton X-100) and then incubated for 45 min at room temperature with secondary antibodies diluted 1:500 in PBS: Alexa Fluor 488 conjugated donkey anti-mouse IgG for GFAP (A-21202, Thermofisher: Waltham, MA, USA), Alexa Fluor 594 conjugated donkey anti-rabbit IgG for Iba-1 (A-21207, Thermofisher: Waltham, MA, USA), or Alexa Fluor 488 donkey anti-sheep IgG for CGRP (A-11015, Thermofisher: Waltham, MA, USA). Slides were rinsed 3 × 10 min in PBS, dipped in deionized water, and cover slips mounted using Fluoroshield Mounting Medium with 4′,6-diamidino-2-phenylindole to visualize nuclei (ab104139, Abcam: Cambridge, UK). Tissue sections were imaged using a Leica Microsystems DMI6000B fluorescence microscope and DFC360FX camera with Leica Advanced Application Suite software (Version 2.7.0-9329, Leica Microsystems: Wetzlar, DEU). A region of interest (ROI) was manually defined to contain lamellae 1-4 of the spinal cord dorsal horn using ImageJ software. The dorsal horn was differentiated from surrounding white mater based on brightfield images. Integrated density of fluorescence within the ROI was used to quantify astrocyte/microglia density, and CGRP-immunoreactivity.

### Serum analysis by multiplex assay

At euthanasia, blood was obtained by cardiac puncture, coagulated for 30 min at room temperature, and centrifuged at 3000 rpm for 10 min at 4 °C to collect serum. Serum (*n* = 5–6 mice per diet/per timepoint) was diluted 2-fold in DPBS and analyzed using the MILLIPLEX Mouse Cytokine/Chemokine 32-plex kit (Millipore, St. Charles, MO, USA) on the Luminex™ 200 system (Luminex, Austin, TX, USA) by Eve Technologies Corp. (Calgary, Alberta). The multiplex assay quantified Eotaxin, G-CSF, GM-CSF, IFNγ, IL-1α, IL-1β, IL-2, IL-3, IL-4, IL-5, IL-6, IL-7, IL-9, IL-10, IL-12 (p40), IL-12 (p70), IL-13, IL-15, IL-17, IP-10, KC, LIF, LIX, MCP-1, M-CSF, MIG, MIP-1α, MIP-1β, MIP-2, RANTES, TNFα, and VEGF. The assay sensitivities range from 0.3 to 30.6 pg/mL.

### Statistical analysis

For all assays except histopathological scoring of the joints, outcome measures for mice within each time point were compared between the different diet groups by one-way ANOVA with Tukey’s multiple comparisons test. For histopathological analysis, within each timepoint scores for mice were compared between the different diet groups by non-parametric Kruskal-Wallis test with Dunn’s multiple comparison test. *P < 0.05* was considered significant. Statistical analysis was conducted using GraphPad Prism 8 (Graphpad Software: San Diego, CA, USA).

To assess the effect of diet, adiposity, knee OA, and IVD degeneration on behavioral, molecular, and histological changes assessed, bivariate and multivariate linear regression models were used to identify which variables remained independently associated with the other outcomes. Bivariate and multivariate modeling was conducted using STATA 16 (StataCorp LLC: College Station, TX, USA).

## Results

### Weight and adiposity

**Fig. 1:**
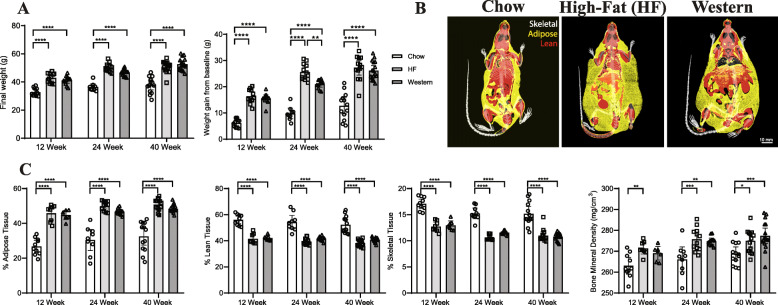
Chronic consumption of the high-fat and western diets increases adiposity in C57BL/6N mice. **a** At all timepoints, mice fed the high-fat and western diets showed significant increases in overall weight and in weight-gain from baseline compared to age-matched chow-fed controls. **b** Representative reconstructed micro-CT images of mice following 40 weeks of experimental diets. Isotropic surface-rendering of skeletal tissue (indicated in white) is overlaid with a mid-coronal slice where lean tissue is indicated in red and adipose tissue is indicated in yellow. **c** Quantitative micro-CT analysis of whole-body composition showed a significant increase in adiposity and significant decreases in both percentage of lean and skeletal and in mice fed the high-fat and western diet mice compared to age-matched chow-fed controls at all time points. A significant increase was also seen in bone mineral density in mice following consumption of the high-fat and western diets at the 24- and 40-week timepoints. *n* = 9–16 mice per timepoint, per diet. Data are displayed as mean ± 95% CI; data points for each mouse are graphed within each group. *P < 0.05, **P < 0.01, ***P < 0.001, ****P < 0.0001 by 2-way ANOVA

As expected, following 12, 24, and 40 weeks mice fed the high-fat and western diets showed a significant increase in body mass and weight gain compared to age-matched chow-fed controls (Fig. 1a). Analysis of body composition by micro-CT (Fig. 1b) demonstrated that the increase in adiposity in mice fed the experimental diets was accompanied by a significant decrease in the percentage of both lean and skeletal tissues at all time points (Fig. 1c). This analysis also showed a significant increase in overall bone mineral density (BMD) in mice fed the high-fat diet at all timepoints, and at the 24- and 40-week timepoints for mice fed the western diet, compared to age-matched chow-fed controls (Fig. 1c).

### Behavioral indicators of axial discomfort

**Fig. 2:**
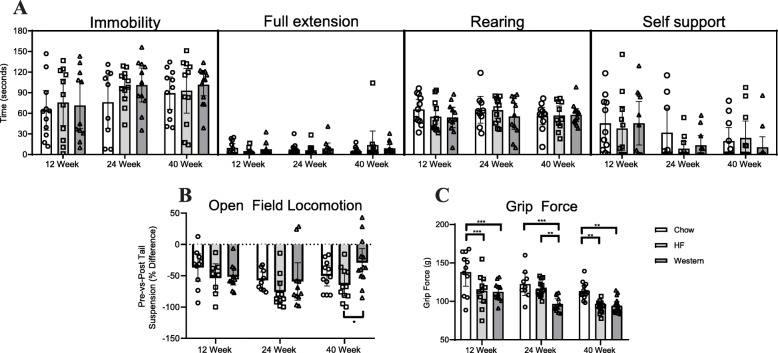
Diet-induced obesity reduces grip strength but does not alter behavior in tail suspension. **a** During tail suspension, the duration of time spent by mice immobile, in full extension, rearing or self-supported was quantified. No significant differences were detected between diet groups at any of the time points assessed. **b** Stretch-evoked discomfort was assessed using the open field assay, in which the total distance covered in 5 min immediately before (pre) and after (post) the 3 min tail suspension assay was quantified. Obesity induced by the high-fat and western diets did not alter behavior of mice in open field compared to age-matched chow-fed controls at any of the time points assessed. However, a significant difference was seen between mice fed a high-fat and western diet at the 40-week timepoint. **c** Grip force during axial stretch was reduced in obese mice. Mice fed the high-fat diet showed a significant decrease in grip force at the 12- and 40-week time points compared to age-matched chow-fed controls. Mice fed the western diet showed a significant decrease in grip force compared to age-matched chow-fed controls at all time points. *n* = 9–16 animals per timepoint, per diet. Data are plotted mean ± 95% CI; data points for each mouse are graphed within each group. *P < 0.05, **P < 0.01, ***P < 0.001 by 2-way ANOVA

We first investigated whether mice fed the high-fat or western diet showed behavioral indicators of stretch-induced axial discomfort using three complimentary assays established as indicators of discogenic back pain in a mouse model of degeneration [40]: behavior during tail suspension, changes in spontaneous activity after tail suspension, and tolerance to axial stretching in the grip force assay. In the tail suspension test, no significant differences were observed between diet groups at any time point investigated (Fig. 2a). Similarly, changes in spontaneously activity induced by the tail suspension assay (locomotion pre versus post tail suspension) were not altered between experimental diet groups and age-matched chow-fed controls (Fig. 2b). Grip force during axial stretch was reduced in mice fed the high-fat diet compared to age-matched chow-fed controls at 12 and 40 weeks and in mice fed the western diet at all time points compared to control (Fig. 2c), suggesting decreased tolerance to axial stretch.

### Behavioral indicators of mechanical and cold sensitivity

**Fig. 3:**
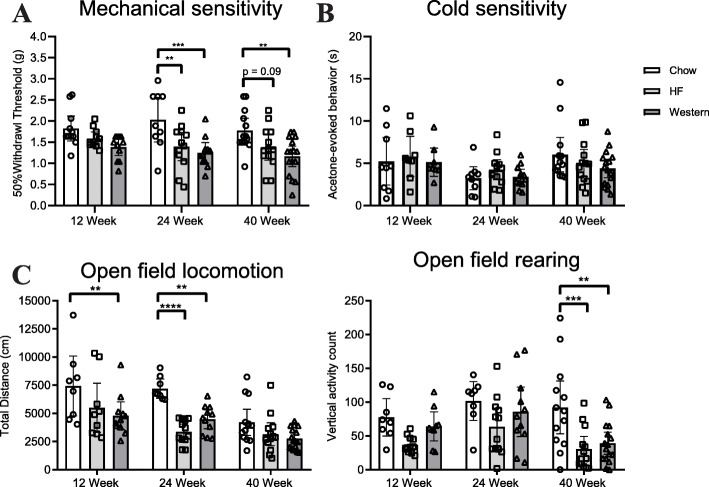
Diet-induced obesity increases sensitivity to mechanical stimulation and alters spontaneous locomotion. **a** Mechanical sensitivity of the hind paw was assessed by manual application of Von Frey filaments using the Chaplin up-down method. Mice fed the western diet showed a significant decrease in withdrawal threshold at the 24- and 40-week time points compared to age-matched chow-fed controls, indicative of increased mechanical sensitivity. Mice fed a high-fat diet showed a significant decrease in withdrawal threshold at the 24-week timepoint compared to control. **b** Sensitivity to cold was assessed by measuring the time spent in behavior evoked by evaporative cooling of acetone (flicking, stamping, or licking of ventral surface of the paw) during the first 40 s following application of acetone to the ventral surface of the hind paw. No significant differences were seen between the diet groups at any timepoint. **c** Spontaneous locomotor activity was recorded over three 2-h sessions and averaged. Mice fed the western diet showed a significant decrease in the total distance traveled at the 12- and 24-week timepoint compared to age-matched chow-fed controls, while mice fed the high-fat diet showed a decrease at the 24-week timepoint. The number of rearing events was significantly decreased in mice fed the high-fat and western diets compared to controls at the 40-week timepoint. *n* = 9–16 animals per timepoint, per diet. Data are plotted mean ± 95% CI; data points for each mouse are graphed within each group. *P < 0.05, **P < 0.01, ***P < 0.001, ****P < 0.0001 by 2-way ANOVA

Mechanical and cold sensitivity were assessed in the hind paw using the Von Frey assay and by measuring the response of mice to the evaporative cooling of acetone, respectively. Mice fed the western diet showed a significant increase in mechanical sensitivity at the 24- and 40-week time points compared to age-matched chow-fed controls, while mice fed a high-fat diet showed a significant increase in sensitivity only at the 24-week timepoint compared to controls (Fig. 3a). In contrast, no significant difference was observed in sensitivity to cold between mice in either experimental diet group compared to age-matched chow-fed controls at any time point (Fig. 3b).

### Spontaneous locomotion

Behavior and locomotion in open field was assessed for all mice over a 2 h period. Mice fed the western diet showed a significant decrease in total distance traveled following 12 and 24 weeks compared to age-matched chow-fed controls. Mice fed the high-fat diet showed a significant reduction in locomotion following 24 weeks compared to age-matched controls (Fig. 3c). The number of rearing events was also significantly decreased in mice fed the high-fat and western diets at the 40-week time point compared to age-matched chow-fed controls (Fig. 3c).

### Assessment of IVD degeneration

**Fig. 4:**
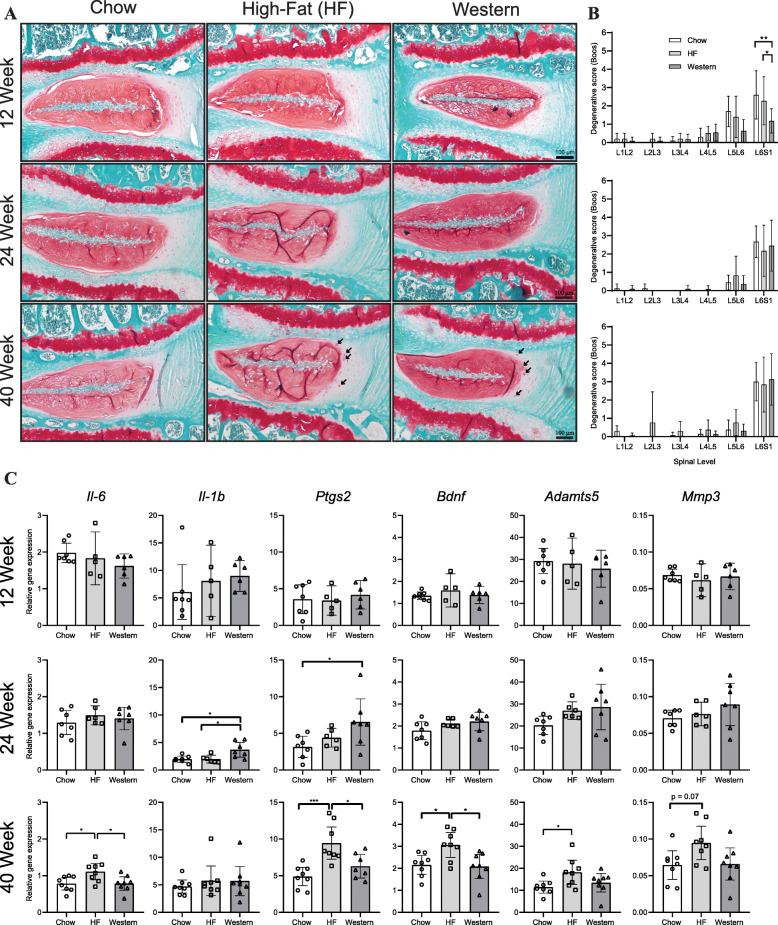
Effect of diet-induced obesity on the intervertebral disc. **a** Representative histological sections of the L6/S1 spinal level of the lumbar spine stained with safranin-o/fast green from mice fed control chow, high-fat, or western diet for 12, 24, or 40 weeks. The accumulation of hypertrophic cells was detected within the inner annulus fibrosus of mice fed the high-fat and western diets for 40 weeks (highlighted by black arrows). **b** Evaluation of the grade of histopathological IVD degeneration using the modified Boos scoring system showed no significant differences between mice fed the control chow, high-fat, or western diets at the 24- and 40-week timepoints. At the 12-week timepoint, mice fed the western diet showed a significant decrease in the degenerative score compared to mice fed chow and high-fat diets. *n* = 9–16 animals per timepoint, per diet. Data are analyzed by Kruskal-Wallis test. **c** SYBR-based qPCR of intact thoracic intervertebral discs showed no significant difference between mice fed a chow, high-fat, or western diet at the 12-week timepoint for any genes investigated. At the 24-week timepoint, significant increases in *Il-1b* and *Ptgs2* expression were seen in mice fed the western diet compared to control. By 40 weeks, significant increases in *Il-6, Ptgs2, Bdnf,* and *Adamts5* expression were seen in mice fed the high-fat diet compared to control. *n* = 5–8 animals per diet/per timepoint. Analyzed by one-way ANOVA. All data are plotted mean ± 95% CI; data points for each mouse are graphed within each group. *P < 0.05, ***P < 0.001

The effects of the high-fat and western diets on IVD health were assessed using both histopathological evaluation and molecular analysis (Fig. 4). On average, no overt differences were detected in the histological appearance of lumbar IVDs between mice fed either the high-fat or western diet for 12 and 24 weeks compared to age-matched chow-fed controls (Fig. 4a). Accordingly, histopathological scoring using the modified Boos system showed no significant differences in degeneration between the groups. However, when data was analyzed by individual spinal level, mice fed the western diet showed significantly lower scores than their high-fat or chow-fed counterparts at the L6S1 spinal level at the 12-week timepoint (Fig. 4b). Following 40 weeks on the experimental diets, an accumulation of hypertrophic cells surrounded by a glycosaminoglycan-rich pericellular matrix was consistently detected in the inner annulus fibrosus of mice fed both the high-fat and western diets, but not in age-matched chow-fed control mice (Fig. 4a—black arrows). Despite this observation, histopathological scoring revealed no significant degeneration in the diet groups compared to chow-fed controls at the 40-week time point (Fig. 4b).

qPCR was used to quantify expression of markers of inflammation, neural ingrowth, and matrix degrading enzymes in thoracic IVDs to further investigate molecular changes associated with diet-induced obesity. Mice fed the high-fat or western diets showed no significant differences in the expression of the genes investigated at the 12-week timepoint compared to age-matched chow-fed controls (Fig. 4c). Mice fed the high-fat diet showed no significant differences in gene expression compared to chow-fed controls at the 24-week timepoint for any of the genes investigated. At the 40-week timepoint, mice fed the high-fat diet showed increased expression of inflammatory mediators (*Il-6*, *Ptsg2),* neurotrophins (*Bdnf*), and matrix degrading enzymes (*Adamts5)* compared to age-matched chow-fed controls (Fig. 4c). Mice fed the western diet showed increased expression of the inflammatory mediators *Il-1b* and *Ptgs2* compared to chow-fed controls at 24 weeks; however, no significant differences in gene expression were detected at the 40-week timepoint (Fig. 4c).

### Assessment of degenerative changes in the knee

**Fig. 5:**
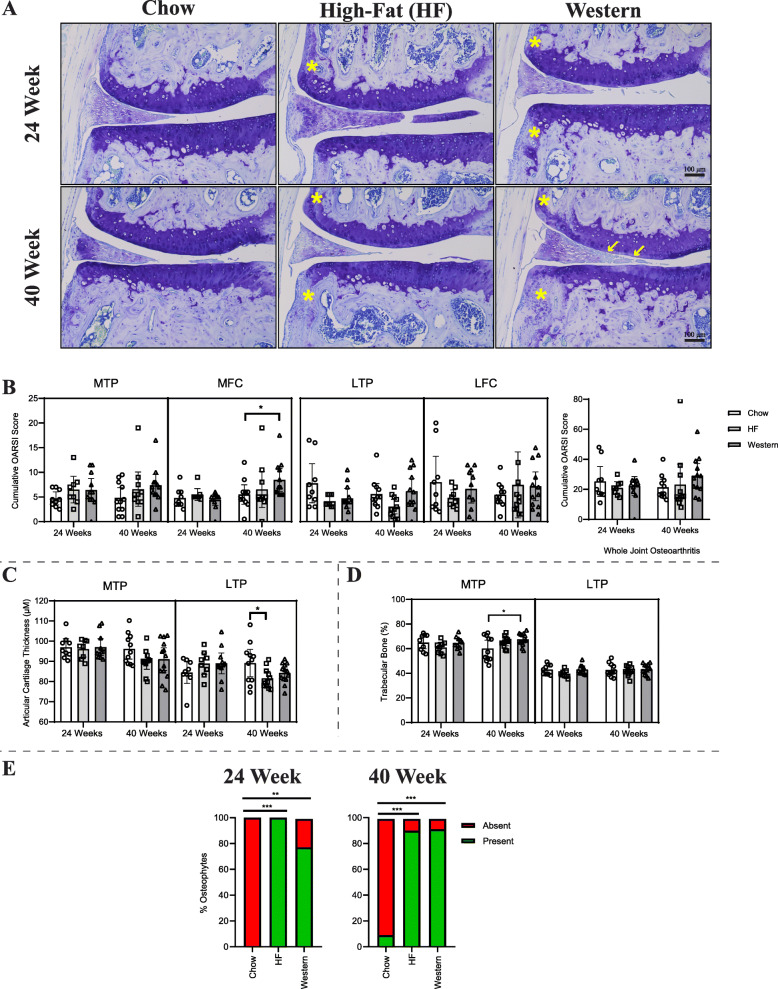
Effect of diet-induced obesity on the knee joint. **a** Representative histological coronal sections of the medial knee compartment stained with toluidine blue from mice fed either control chow, high-fat, or western diet for 24 or 40 weeks. Images are oriented with the medial femoral condyle (MFC) located superiorly, and the medial tibial plateau (MTP) inferiorly. White arrows indicate a loss of proteoglycan staining and focal fibrillation of the cartilage, and yellow asterisks indicate osteophyte formation. **b** Histopathological grading of the knee joints using the murine Osteoarthritis Research Society International (OARSI) scale corresponding to MTP, MFC, lateral tibial plateau (LTP), and lateral femoral condyle (LFC), combined to generate the summed score for the whole joint. Mice fed the western diet for 40 weeks showed a significant increase in the degenerative score in the MFC compared to those fed the control chow. However, no difference was seen in the whole joint score between any of the groups at either timepoint. **c** Average articular cartilage thickness. After 40 weeks on the high-fat diet, mice presented with decreased articular cartilage thickness on the LTP. No other differences were seen in any other joint compartment. Data analyzed by Kruskal-Wallis test. **d** Percent trabecular bone in the medial and lateral subchondral compartments of the tibia. Mice fed  the western diet for 40 weeks exhibited significantly more trabecular bone in the medial compartment for the tibia. Analyzed by one-way ANOVA. **e** Presence and absence of osteophytes was assessed in all mice. Mice fed the high-fat and western diets showed increased osteophyte formation compared to chow-fed controls at both timepoints. Analyzed by Kruskal-Wallis test. *n* = 9–16 animals per diet/per timepoint. All data are plotted mean ± 95% CI, *P < 0.05, **P < 0.01, ***P < 0.001

Since diet-induced obesity leads to arthropathies such as knee OA [23], we investigated degenerative changes to the knee joint as a potential contributor to the pain-related behavioral outcomes assessed. We focused this analysis on the 24- and 40-week timepoints where changes in behavioral measures were most consistently identified. No overt histological differences were detected in the knee joints of mice fed either the high-fat or western diet for 24 weeks compared to age-matched chow-fed controls (Fig. 5a). Histopathological scoring using the OARSI system supported these observations (Fig. 5b). At the 40-week timepoint, mice fed the western diet showed focal areas of decreased proteoglycan staining in the medial femoral condyle (MFC) (Fig. 5a), resulting in significantly higher OARSI scores compared to age-matched controls (Fig. 5b). No significant differences were detected in the other compartments of the knee joint or in the cumulative OARSI scores for the whole joint.

**Fig. 6:**
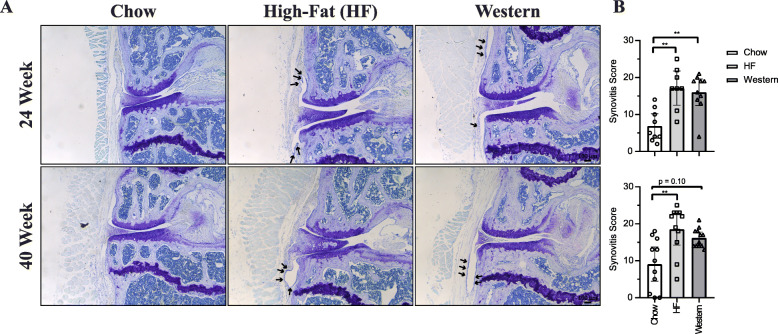
Effect of diet-induced obesity on synovial inflammation and hyperplasia. **a** Representative coronal sections of the medial compartment of the knee stained with toluidine blue from mice fed either control chow, high-fat, or western diets for 24 or 40 weeks. Images are oriented with medial femoral condyle (MFC) located superiorly, and the medial tibial plateau (MTP) inferiorly. Black arrows indicate synovial hyperplasia. **b** Histopathological scoring assessed indicators of synovial hyperplasia and inflammatory infiltration. Summed scores represent the sum of the medial and lateral compartments scores across 7 serial sections. Mice fed a high-fat diet showed a significant increase in synovial inflammation and hyperplasia compared to chow-fed control at both the 24 and 40 week timepoint, while mice fed a western diet showed a significant increase in synovitis at only the 24-week timepoint. *n* = 8–12 animals per diet/per timepoint. Analyzed by Kruskal-Wallis test. All data are plotted mean ± 95% CI, **P < 0.01

In addition to histopathological analysis, histomorphometry was used to assess morphological changes in the knee. Although no significant differences in cartilage thickness were detected in mice following 24 weeks on the experimental diets, mice fed the high-fat diet for 40 weeks showed a significant decrease in cartilage thickness compared to age-matched chow-fed controls, specific to the lateral femoral condyle (Fig. 5c). Mice fed the western diet for 40 weeks showed a significant increase in subchondral trabecular bone in the medial tibial plateau (measured as the area of trabecular bone between the articular cartilage and growth plate) compared to age-matched chow-fed controls, suggesting sclerosis of the subchondral bone (Fig. 5d). Mice fed the high-fat and western diet for 24 or 40 weeks also showed a significant increase in osteophyte formation compared to chow-fed controls (Fig. 5e). Lastly, a significant increase in synovial hyperplasia and inflammatory infiltration, hallmarks of synovitis, were detected in mice fed the high-fat diet at the 24- and 40-week timepoints and in mice fed the western diet at the 24-week timepoint compared to chow-fed controls (Fig. 6).

### Analysis of sensory neuroplasticity within the lumbar spinal cord

**Fig. 7:**
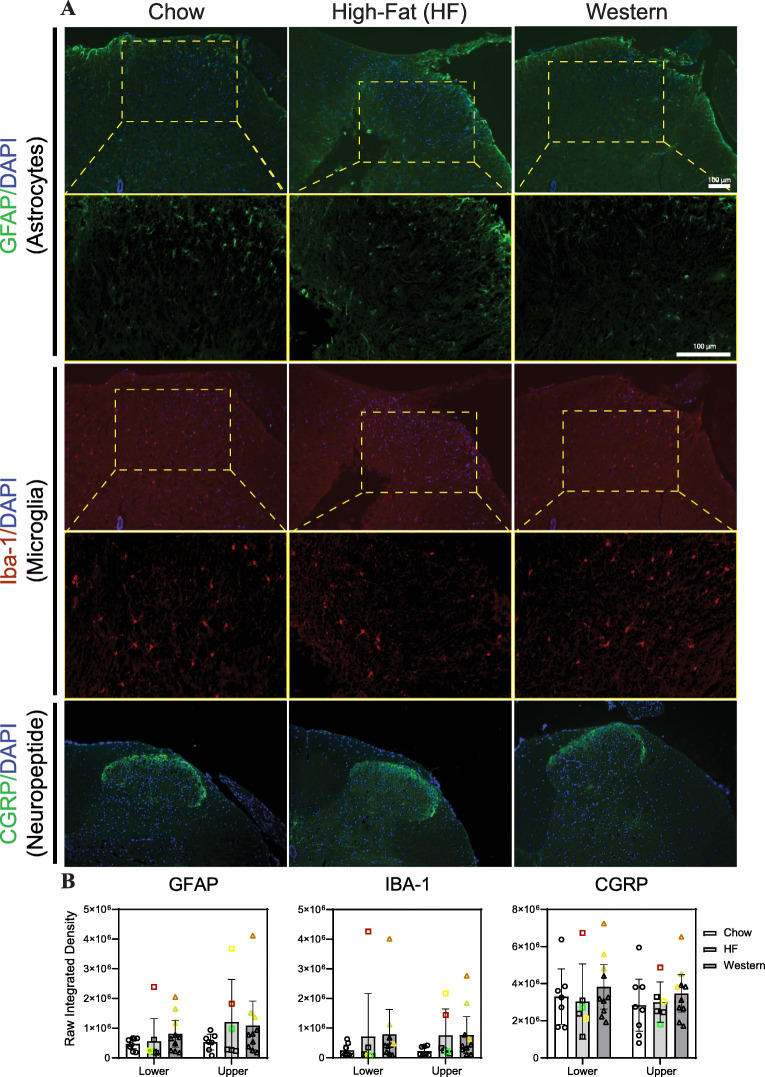
Effect of diet-induced obesity on neuroplastic changes within the lumbar spinal cord. **a** Representative images showing transverse sections of the dorsal horn of the lumbar spinal cord used for immunohistochemical analysis of neuroplastic changes associated with chronic pain. Tissues were isolated from mice following 40 weeks of experimental diet. Slides were stained for glial fibrillary acidic protein (GFAP), ionized calcium-binding adapter molecule 1 (IBA1), and calcitonin gene-related peptide (CGRP). Yellow boxes indicate the region of interest for high magnification images (second row) for GFAP and IBA-1. (**b**) The fluorescence intensity was averaged across the region of interest (lamellae 1-4 of the dorsal horn) of the upper (L1-L2) and lower (L3-L6) lumbar spinal cords. Mice fed a high-fat or western diet for 40 weeks showed no significant differences in immunoreactivity for any of the proteins investigated. *n* = 6–8 animals/group. Individual data points of the same color indicate the same animal. Data are plotted mean ± 95% CI

To assess neuroplastic changes associated with neuroinflammation and chronic pain, lumbar spinal cords from mice at the 40-week time point were assessed for markers of astrocytes (glial fibrillary acidic protein, GFAP), microglia (ionized calcium-binding adapter molecule 1, IBA-1), and nociceptive innervation (calcitonin gene-related peptide, CGRP; Fig. 7a). Although multiple mice in both the high-fat and western diet groups showed increased GFAP and IBA-1 staining in the upper and lower lumbar spinal cord compared to the average values for chow-fed controls, quantification of fluorescent intensity within the dorsal horn showed no significant difference between diet conditions for any of the proteins investigated (Fig. 7b).

### Circulating inflammatory factors

Luminex xMAP multiplex assays were used to quantify a panel of 32 cytokines, chemokines, and growth factors in the serum of experimental mice. Mice fed the western diet showed increased levels of interleukin (IL)-1β, IL-6, IL-10, and tumor necrosis factor alpha (TNFα) at the 12- and 40-week timepoints; however, no significant differences were seen when compared to chow-fed control due to variability between animals (Table 1). Despite this variability, at the 40-week timepoint, a significant increase in circulating monocyte chemoattractant protein 1 (MCP-1) was observed in mice fed a western diet compared to chow-fed controls (Table 1). Animals fed a western diet for 24 weeks also showed significantly lower levels of circulating eotaxin and IL-1α compared to chow-fed controls, but no differences in any of the other cytokines investigated (Supplementary Table 3).

**Table 1:** Multiplex analysis of cytokines, chemokines, and growth factors in serum

Analyte	12 Week				24 Week				40 Week			
	Chow, ***n*** = 6	HF, ***n*** = 6	Western, ***n*** = 6	***p*** value	Chow, ***n*** = 5	HF, ***n*** = 6	Western, ***n*** = 6	***p*** value	Chow, ***n*** = 5	HF, ***n*** = 6	Western, ***n*** = 6	***p*** value
	Mean (SD)	Mean (SD)	Mean (SD)		Mean (SD)	Mean (SD)	Mean (SD)		Mean (SD)	Mean (SD)	Mean (SD)	
IL-1B	13.9 (17.5)	32.3 (58.6)	39.4 (43.8)	0.54	22.5 (26.9)	15.3 (15.7)	41.1 (89.3)	0.72	2.0 (1.8)	2.0 (1.4)	6.4 (6.7)	0.16
IL-6	17.2 (14.3)	20.9 (28.1)	47.6 (67.0)	0.43	25.7 (31.9)	28.7 (35.0)	13.6 (12.2)	0.63	2.7 (3.8)	4.1 (1.9)	20.0 (30.2)	0.24
IL-10	45.8 (51.4)	56.6 (85.1)	153 (261.2)	0.47	19.0 (24.1)	17.2 (18.7)	15.1 (18.7)	0.95	0.5 (1.0)	0.4 (0.9)	2.1 (2.9)	0.25
IP-10	129.4 (53.8)	122.0 (32.4)	139.5 (38.7)	0.78	90.9 (53.2)	105.9 (43.8)	63.6 (49.5)	0.34	106 (14.3)	113.7 (24.2)	144.7 (67.9)	0.32
KC	453.4 (323.3)	690.2 (843.0)	856.0 (1324)	0.75	527.2 (363.1)	452.0 (230.1)	240.7 (108.5)	0.17	191.1 (85.0)	355.3 (223.4)	258.8 (139.5)	0.28
MCP-1	53.7 (58.7)	51.6 (46.4)	78.3 (65.6)	0.68	54.3 (23.8)	68.1 (42.3)	165.2 (259.5)	0.45	**13.6 (9.6)**	**63.8 (39.1)**	135.3 (93.7) *#	0.02*
TNFa	28.4 (31.4)	37.8 (54.2)	41.9 (35.0)	0.85	23.9 (23.6)	16.0 (22.7)	53.4 (96.2)	0.54	1.0 (1.9)	2.2 (4.1)	3.0 (5.1)	0.63
VEGF	2.2 (1.87)	6.8 (14.2)	13.1 (27.8)	0.59	2.9 (4.2)	1.5 (2.5)	1.9 (2.1)	0.73	5.4 (8.4)	2.1 (3.1)	1.0 (0.5)	0.33

Values are displayed in pg/mL and analyzed by one-way ANOVA. *P* < 0.05 is significant

*Significantly different from chow diet by Tukey's post hoc test

^#^Significantly different from HF diet by Tukey’s post hoc test

### Linear regression analysis

To directly examine associations in the context of the variability observed within each experimental group in our study, linear regression modeling was conducted to determine whether diet, adiposity, or age were predictors of behavioral, histological, or systemic outcomes (Table 2). Bivariate modeling demonstrated that both adiposity and age are independent predictors of multiple indicators of pain including stretch-induced axial discomfort (grip force), mechanical sensitivity (Von Frey assay), and spontaneous locomotion (open field). Despite their association with behavioral alterations, neither adiposity nor age was associated with measures of joint degeneration or most circulating factors assessed (Table 2). However, synovitis and systemic TNFα were associated with adiposity (bivariate/ multivariate) and age (bivariate), respectively (Table 2). When diet, adiposity, and age were accounted for, age was found to be the most robust predictor of all outcomes measured (Table 2). The multivariate model also showed that diet and adiposity are covariates for grip force and rearing in open field, respectively (Table 2). To determine if histopathological scores for joint damage were independently associated with behavioral indicators of pain, bivariate and multivariate regression modeling was completed. In this study, no significant association was detected between histopathological scores for IVD degeneration and pain-related behaviors. A weak but significant association was detected between histopathological scores for knee OA and grip force, but not for any of the other behavioral metrics assessed (Supplementary Table 4). Synovitis was significantly associated with alterations in spontaneous activity, including distance traveled, rest time, and rearing in open field (Supplementary Table 4).

**Table 2:** Impact of diet-induced obesity and age on behavioral, molecular, and histological changes

Parameter	Bivariate (***r***)		Multivariate (***β***, ***r***^**2**^)				
	% Adipose	Timepoint	Diet (β)		% Adipose (***β***)	Timepoint (***β***)	Whole model (***r***^**2**^)
			Experimental (Obesogenic)	Western			
**Behavioral**							
Grip force	− 0.41***	− 0.45***	− 21.96**	− 6.23	0.4	− 0.841**	0.45***
Mechanical sensitivity (Von Frey)	− 0.42***	− 0.21*	− 0.35	− 0.14	− 0.0007	− 0.006	0.26***
Cold sensitivity (Acetone)	0.04	0.01	–	–	–	–	0.08
Tail suspension							
Rearing	0.01	0.11	–	–	–	–	0.02
Immobility	0.18	0.19	–	–	–	–	0.05
Self-support	− 0.22*	− 0.30**	1.73	− 0.46	− 0.71	− 0.74*	0.10*
Stretch	0.06	0.10	–	–	–	–	0.07
Open field							
Locomotion	− 0.49***	− 0.35***	− 1226	− 59.3	− 92.7	− 93.5***	0.33***
Rearing	− 0.52***	− 0.40***	− 0.88	− 7.41	− 2.87*	− 2.059***	0.39***
**Histopathology**							
IVD degeneration (average Boos)	0.065	0.04	–	–	–	–	0.03
Knee OA (cumulative OARSI)	0.07	0.08	–	–	–	–	0.05
Synovitis	0.68***	0.102	3.535	− 0.692	0.325*	0.063	0.478***
**Neuro-inflammation**							
Lower spinal cord							
GFAP	0.23	–				–	0.30
IBA-1	0.23	–	–	–	–	–	0.06
CGRP	0.024	–	–	–	–	–	0.18
Upper spinal cord							
GFAP	0.21	–	–	–	–	–	0.08
IBA-1	0.33	–	–	–	–	–	0.12
CGRP	0.31	–	–	–	–	–	0.18
**Systemic factors**							
IL-1B	0.04	0.21	–	–	–	–	0.12
IL-6	0.06	0.22	–	–	–	–	0.09
IL-10	0.03	0.21	–	–	–	–	0.14
IP-10	0.03	0.07	–	–	–	–	0.001
KC	0.002	0.25	–	–	–	–	0.08
MCP-1	0.23	0.04	–	–	–	–	0.18
TNFa	0.03	0.30*	–	–	–	–	0.16
VEGF	0.01	0.12	–	–	–	–	0.06

Experimental/obesogenic diets include the high-fat and western diet

*P < 0.05, **P < 0.01, ***P < 0.001

## Discussion

Obesity is one of the largest modifiable risk factors for IVD degeneration and LBP [35, 36], yet the biological mechanisms underlying this association are unknown. The current study was designed to investigate the longitudinal effects of diet-induced obesity on inflammation, IVD degeneration, knee OA, sensory neuroplasticity, and pain using the mouse as a pre-clinical model. We show that obesity induced by both a high-fat and high-fat/high-sugar western diet led to behavioral indicators of pain in as little as 12 weeks, preceding gross structural changes to the IVD and articular cartilage of the knee. Following 40 weeks, changes in cellular morphology within the inner AF of the IVD were detected in mice fed both obesogenic diets compared to chow-fed controls; however, these changes were not associated with increased histopathological degeneration. Chronic consumption of the high-fat diet was associated with increased expression of *Il-6*, *Ptgs2*, *Bdnf*, *Adamts-5,* and *Mmp-3* within the IVD, a decrease in articular cartilage thickness, osteophyte formation, and synovitis. In contrast, chronic consumption of the western diet was associated with increased expression of *Il-1b* and *Ptgs2* within the IVD, histopathological features of early OA, osteophyte formation, synovitis, subchondral bone sclerosis, and increased serum MCP-1 levels. These findings highlight the complex interplay between diet, adiposity, pain, inflammation, and joint health.

Rodent models are useful to study IVD biology since they allow insight into biological processes that regulate tissue homeostasis and degeneration, yet few studies have investigated the association between IVD degeneration and clinically relevant pain [47]. Given the discordance between structural IVD degeneration and pain in humans [10, 48, 49] and pre-clinical models [50], it is important to investigate pain, structural IVD degeneration, and their association. Several indirect, quantitative behavioral assays have been developed to evaluate pain-like behaviors in animals for a variety of different pain states [51, 52]. Many of these metrics are not specific to back pain but are used to assess joint, inflammatory, and neuropathic pain [53, 54]. However, stretch-induced axial discomfort (measured by grip force and tail suspension assays) is established as a reliable measure of axial back pain in mice [40]. In the current study, mice fed obesogenic diets showed significant impairments in grip force at all timepoints compared to control mice, suggesting axial discomfort. In contrast, no difference was detected in tail suspension between groups. This potentially contradictory data may be influenced by the nature of the tail suspension assay, which was originally developed for depressive behavior in mice [55], since obesity is also associated with depression [33]. In fact, recent studies demonstrated that mice fed a high-fat diet for 8 weeks showed increased immobility in tail suspension, interpreted as depression-like behavior [56]. In addition to changes in body mass that may impact activity in suspension, this confounding factor may impact the outcome and interpretation of our findings in tail suspension. Obese mice also displayed mechanical but not thermal (cold) hypersensitivity of the hind paw. These alterations are consistent with reports of surgically induced IVD degeneration in rats [57] yet contrast the SPARC-null mouse model of IVD degeneration which shows thermal but not mechanical hypersensitivity [39]. While the mechanisms underlying these differences are unclear, mechanical sensitivity is common in both inflammatory and neuropathic pain [58], and different models of pain likely impact nociception through different mechanisms [59]. Obesity also altered non-reflexive (spontaneous) behaviors including distanced traveled and rearing in open field—measurements that have been shown to decrease in both inflammatory and neuropathic pain models [60].

Previous studies have established that high-fat diet-induced obesity accelerates OA progression in mice [23, 26]; however, pain-related behaviors were independent of OA severity [23]. As IVD degeneration is associated with pain-related behaviors common to OA [38], we characterized both IVD degeneration and knee OA in our experimental mice. Histopathological scoring of lumbar IVDs did not reveal significant degeneration caused by the high-fat or western diets. However, mice fed both obesogenic diets showed an accumulation of hypertrophic cells in the inner annulus fibrosus at 40 weeks, suggesting early degenerative change. Moreover, increased expression of inflammatory mediators (*Il-1b*, *Il-6, Ptgs2*), matrix degrading enzymes (*Adamts5*) and neurotrophins (*Bdnf*) were detected in the IVDs of mice fed the high-fat and western diets. These inflammatory cytokines drive IVD pathogenesis associated with ECM degeneration and expression of neurogenic factors such as NGF and BDNF that contribute to pain [61]. Similarly, moderate histopathological degeneration articular cartilage was detected in the knee at the 40-week timepoint in mice fed the western diet. Linear regression modeling indicates that the behavioral indicators of pain assessed are independent of joint degeneration, except for the grip force assay where knee OA was a significant predictor of impairment. Together these findings suggest that chronic diet-induced obesity may accelerate the progression of IVD degeneration and knee OA; however, these changes are mild and likely independent of most pain-related behaviors.

Our findings suggesting that pain-related behaviors precede molecular and structural degradation of joint tissues raise questions related to the source of the pain observed. These findings are consistent with the hypothesis that pain is multifactorial. Obesity is a state of chronic inflammation associated with increases in circulating inflammatory cytokines including TNFα and IL-6 in humans [62]. Inflammation can contribute to peripheral and central sensitization and may lead to hyper-excitability of the nervous system and chronic pain [63]. While its role in LBP is not well characterized, synovial inflammation is considered a major contributor to OA-related pain [64–66]. We show that consumption of high-fat and western diets led to synovitis (synovial hyperplasia and increased inflammatory infiltration) and that consumption of the western diet led to increased levels of circulating MCP-1, a pro-algesic mediator that can increase primary afferent neuron activity [67, 68]. Linear regression modeling also identified synovitis as a predictor of diminished spontaneous movement. Obesity may also impact central pain processing; in mice, consumption of a high-fat diet increased the activation of microglia [69] while exposure of cultured astrocytes to saturated fatty acids induces cytokine release and astrocyte inflammation [70]. These neuroplastic changes can contribute to central sensitization through multiple mechanisms, including increased release of inflammatory factors contributing to modulation of synaptic activity [63]. Although the averaged values of GFAP and IBA-1 detected in the spinal cord were not significantly different between groups in our study, multiple mice in both the high-fat and western diet groups showed increased activation of both microglia and astrocytes at the 40-week timepoint. These alterations to the nociceptive pathways at either the peripheral or central level may contribute to the pain response seen. Our findings of obesity-induced synovitis and subchondral bone sclerosis also highlight the possibility that other musculoskeletal structures (i.e., joints, muscle, bone, ligaments) may be affected by obesity and contribute to pain.

Although the average weight gain was similar between the two obesogenic diets evaluated in this study, important differences in outcomes were detected. Mice fed the western diet showed a more consistent pain response compared to control at all timepoints than did mice fed the high-fat diet. Furthermore, although synovitis and osteophyte formation were observed for both, cartilage degeneration resulting in histopathological detection of knee OA and systemic inflammation were only detected in mice fed the western diet. These findings highlight the importance of dietary composition. In the context of OA, dietary fatty acid and carbohydrate composition can significantly impact joint health [24, 71]. Diets high in saturated fatty acids or ω-6 polyunsaturated fatty acids (PUFAs) induce more severe metabolic dysregulation and OA progression than diets enriched with ω-3 PUFAs [24]. These findings may explain our results, as the western diet is higher in saturated fats than the high-fat diet. Dietary composition also impacts IVD health. Diets rich in advanced glycation end-products (AGE) precursors accelerate IVD degeneration in mice in parallel with insulin resistance [72–74].

A confounding factor in the interpretation of our findings was the variability for many of the outcomes investigated, including substantial differences in weight gain between mice on both obesogenic diets. Despite controlling for genetics using an inbred strain, susceptibility to diet-induced obesity can be affected by social stress, microbiome composition, and epigenetic mechanisms [75–77]. Previous studies in mice demonstrated that cartilage damage induced by a high-fat diet is proportional to adiposity [23]. In the current study, adiposity did not predict histopathological measures of joint degeneration; however, adiposity and age were predictors of pain-related behaviors. As adiposity does not directly correlate with systemic or neuro-inflammation in the current model, it is important to investigate all aspects of metabolic syndrome (i.e., circulating lipids, glucose, cytokines/adipokines, blood pressure) and their impact on the musculoskeletal and nervous systems in future studies.

## Conclusions

Taken together, this study highlights the complexity of the relationship between obesity, joint degeneration, and pain. Mice fed a high-fat or western diet showed pain-related behaviors that preceded structural joint degeneration in both the IVD and knee. The chronology of these findings may be of clinical importance, as pain may affect the progression of radiographic joint degeneration. While not directly investigated in IVD degeneration, knee pain has been shown to be a predictor of accelerated radiographic OA through inflammation and reduced mobility [78], which is also seen in IVD degeneration [79]. This raises the intriguing possibility that back pain may be both a consequence of and a contributor to structural IVD degeneration in the current model.

## Supplementary information

**Additional file 1: Supplementary Figure 1.** Spontaneous locomotion (continued). Spontaneous Locomotion activity was recorded over three 2 h sessions and averaged. (A) Mice fed the western diet showed a significant decrease in the average movement velocity at all timepoints compared to age-matched chow fed controls, while mice fed the high-fat diet showed a decrease at the 24-week timepoint. (B) The amount of time spent in the anxiety-inducing center area of the open field enclosure was decreased in mice fed the high-fat and western diets compared to controls but not significant at any timepoint. *n* = 9–16 animals per timepoint, per diet. Data are plotted mean ± 95% CI; data points for each mouse are graphed within each group. *P < 0.05, **P < 0.01, ***P < 0.001, ****P < 0.0001 by one-way ANOVA.

**Additional file 2: Supplementary Figure 2**. SYBR-based qPCR of thoracic IVDs (continued). SYBR-based qPCR of intact thoracic intervertebral discs showed no significant difference between mice fed a chow, high-fat or western diet at the 12-week and 24-week timepoint for any genes investigated. At 40-weeks a significant increase was seen in *Mmp12* expression in mice fed a high-fat diet compared to chow control. *n* = 5–8 animals per diet/per timepoint. Analyzed by one-way ANOVA. All data are plotted mean ± 95% CI; data points for each mouse are graphed within each group. *P < 0.05, ***P < 0.001.

**Additional file 3: Supplementary Table 1.** Experimental diet compositions.

**Additional file 4: Supplementary Table 2.** Real-time qPCR primer sequences.

**Additional file 5: Supplementary Table 3.** Multiplex analysis of cytokines, chemokines and growth factors in serum (Continued).

**Additional file 6: Supplementary Table 4.** Association between behavioral indicators of pain and histological joint damage.

## Data Availability

The data associated with the current study is available from the corresponding author on reasonable request.

## Abbreviations

ADAMTS5: A disintegrin and metalloproteinase with thrombospondin motifs 5
AF: Annulus fibrosus
AGEs: Advanced glycation end-products
BDNF: Brain-derived neurotrophic factor
CEP: Cartilage endplate
CGRP: Calcitonin gene-related peptide
CT: Computed tomography
GFAP: Glial fibrillary acidic protein
IBA-1: Ionized calcium-binding adaptor molecule 1
IL-1β: Interleukin-1β
IL-6: Interleukin-6
IVD: Intervertebral disc
LBP: Low back pain
LTP: Lateral tibial plateau
MCP-1: Monocyte chemoattractant protein 1
MMP-3: Matrix metalloproteinase-3
MRI: Magnetic resonance imaging
MTP: Medial tibial plateau
NGF: Nerve growth factor
NP: Nucleus pulposus
OA: Osteoarthritis
OARSI: Osteoarthritis Research Society International
Ptgs2: Prostaglandin-endoperoxide synthase 2
PUFAs: Polyunsaturated fatty acids
SPARC: Secreted Protein Acidic and Cysteine Rich
TGF-β: Transforming growth factor beta
TNF-α: Tumor necrosis factor alpha
