# Supplementary figures and images for: Diet-induced obesity leads to behavioral indicators of pain preceding structural joint damage in wild-type mice
Source: Arthritis Res Ther. 2021 Mar 22;23:93. doi: 10.1186/s13075-021-02463-5 (PMC7983381; doi:10.1186/s13075-021-02463-5)

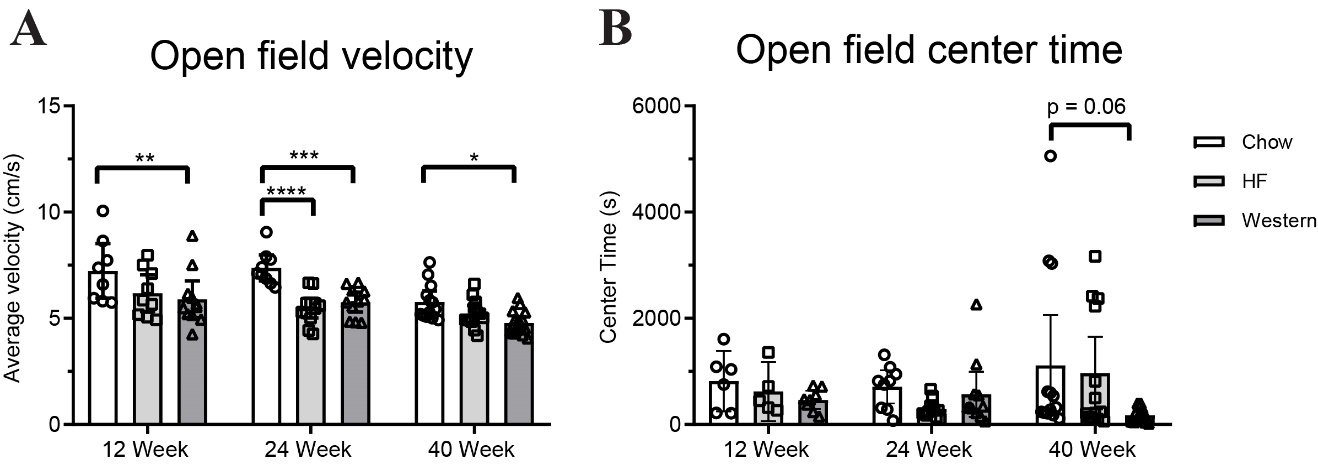

Supplement: Supplementary file 1 — Additional file 1: Supplementary Figure 1. Spontaneous locomotion (continued). Spontaneous Locomotion activity was recorded over three 2 h sessions and averaged. (A) Mice fed the western diet showed a significant decrease in the average movement velocity at all timepoints compared to age-matched chow fed controls, while mice fed the high-fat diet showed a decrease at the 24-week timepoint. (B) The amount of time spent in the anxiety-inducing center area of the open field enclosure was decreased in mice fed the high-fat and western diets compared to controls but not significant at any timepoint. n = 9–16 animals per timepoint, per diet. Data are plotted mean ± 95% CI; data points for each mouse are graphed within each group. *P < 0.05, **P < 0.01, ***P < 0.001, ****P < 0.0001 by one-way ANOVA. [file 13075_2021_2463_MOESM1_ESM.docx]

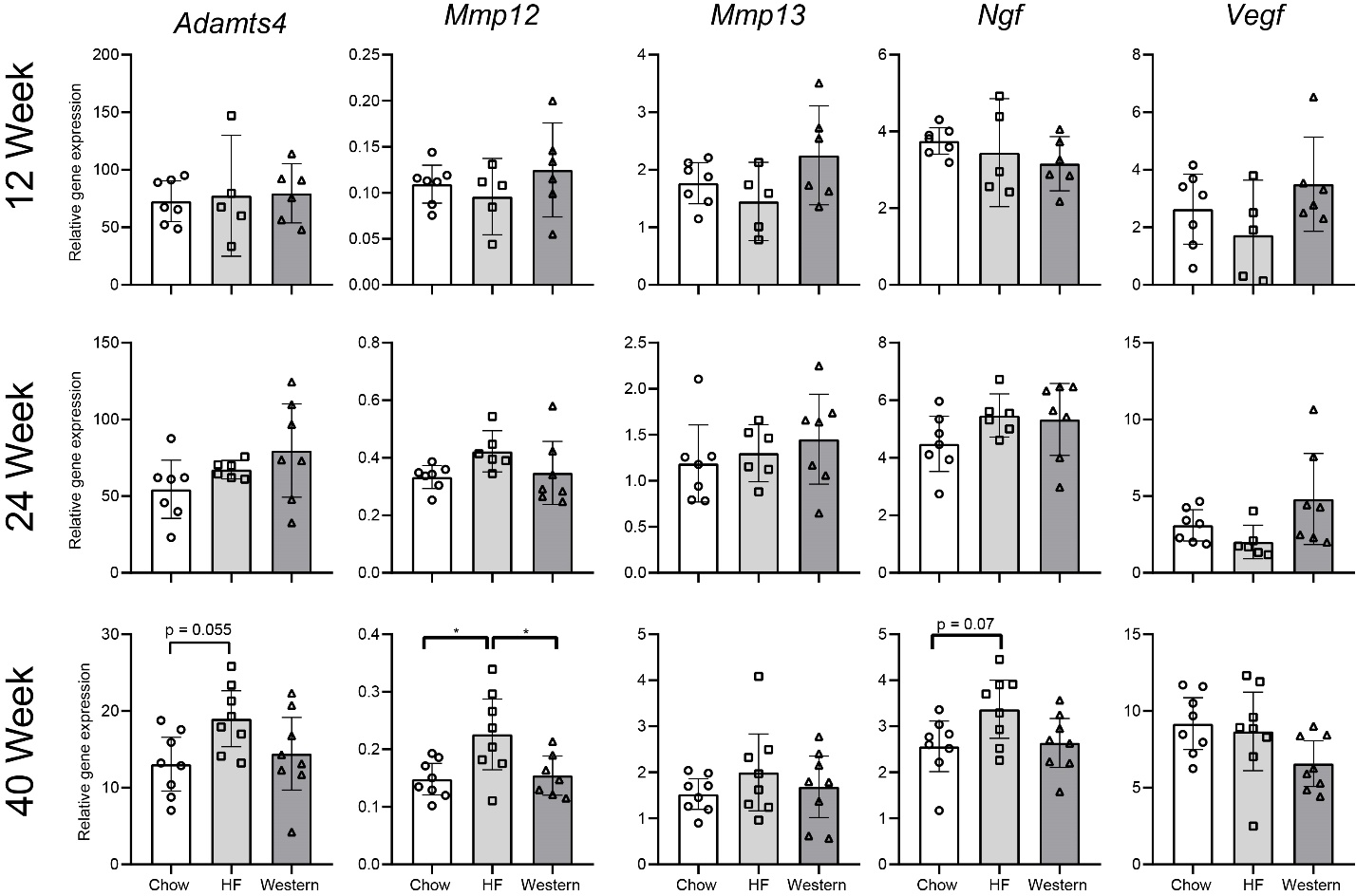

Supplement: Supplementary file 2 — Additional file 2: Supplementary Figure 2. SYBR-based qPCR of thoracic IVDs (continued). SYBR-based qPCR of intact thoracic intervertebral discs showed no significant difference between mice fed a chow, high-fat or western diet at the 12-week and 24-week timepoint for any genes investigated. At 40-weeks a significant increase was seen in Mmp12 expression in mice fed a high-fat diet compared to chow control. n = 5–8 animals per diet/per timepoint. Analyzed by one-way ANOVA. All data are plotted mean ± 95% CI; data points for each mouse are graphed within each group. *P < 0.05, ***P < 0.001. [file 13075_2021_2463_MOESM2_ESM.docx]
