# Supplementary material for: Diet-induced obesity leads to behavioral indicators of pain preceding structural joint damage in wild-type mice
Source: Arthritis Res Ther. 2021 Mar 22;23:93. doi: 10.1186/s13075-021-02463-5 (PMC7983381; doi:10.1186/s13075-021-02463-5)
Supplement: Supplementary file 3 — Additional file 3: Supplementary Table 1. Experimental diet compositions. [file 13075_2021_2463_MOESM3_ESM.docx]

|  | **Control diet** | |  | **Obesogenic diets** | | | |
| --- | --- | --- | --- | --- | --- | --- | --- |
|  | **Chow** | |  | **High-fat** | | **Western** | |
| **Product #** | **Envigo 2018** | |  | **Envigo TD.06414** | | **Envigo TD.10885** | |
| **Content** | **g** | **kcal%** |  | **% by weight** | **kcal%** | **% by weight** | **kcal%** |
| **Protein** | 19.2 | 20 |  | 23.5 | 18.3 | 17.3 | 14.8 |
| **Carbohydrate** | 67.3 | 70 |  | 27.3 | 21.4 | 47.5 | 40.6 |
| **Fat** | 4.3 | 10 |  | 34.3 | 60.3 | 23.2 | 44.6 |
| **kcal/g** |  | 3.85 |  |  | 5.1 |  | 4.7 |
|  |  |  |  |  |  |  |  |
| **Ingredient** | **g/kg** |  |  | **g/kg** |  | **g/kg** |  |
| **Casein** | N/A |  |  | 265 |  | 195 |  |
| **L-Cystine** | N/A |  |  | 4 |  | 3 |  |
| **Corn starch** | N/A |  |  | 0 |  | 55.46 |  |
| **Maltodextrin** | N/A |  |  | 160 |  | 60 |  |
| **Sucrose** | N/A |  |  | 90 |  | 340 |  |
| **Cellulose** | N/A |  |  | 65.5 |  | 50 |  |
| **Soybean oil** | N/A |  |  | 30 |  | 20 |  |
| **Anhydrous Milkfat** | N/A |  |  | 0 |  | 61 |  |
| **Cholesterol** | N/A |  |  |  |  | 1.5 |  |
| **Lard** | N/A |  |  | 310 |  | 0 |  |
| **Vitamin Mix** | N/A |  |  | 21 |  | 19 |  |
| **Mineral Mix** | N/A |  |  | 48 |  | 43 |  |
| **Dicalcium phosphate** | N/A |  |  | 3.4 |  | 0 |  |
| **Choline bitartrate** | N/A |  |  | 3.0 |  | 3.0 |  |
| **SFA (% of fat)** | N/A |  |  | 34.8 |  | 58.3 |  |
| **MUFA (% of fat)** | N/A |  |  | 39.1 |  | 28.2 |  |
| **PUFA (% of fat)** | N/A |  |  | 22.5 |  | 8.9 |  |
|  |  |  |  |  |  |  |  |
| **ω6:ω3 ratio** | N/A |  |  | 13.0 |  | 7.2 |  |
