# Supplementary material for: Diet-induced obesity leads to behavioral indicators of pain preceding structural joint damage in wild-type mice
Source: Arthritis Res Ther. 2021 Mar 22;23:93. doi: 10.1186/s13075-021-02463-5 (PMC7983381; doi:10.1186/s13075-021-02463-5)
Supplement: Supplementary file 4 — Additional file 4: Supplementary Table 2. Real-time qPCR primer sequences. [file 13075_2021_2463_MOESM4_ESM.docx]

| **NCBI Gene Name** | **Primer Sequence 5’ 🡪 3’** |
| --- | --- |
| *Il6* Fwd | TCTCTGCAAGAGACTTCCATCCAGT |
| *Il6* Rev | AGTAGGGAAGGCCGTGGTTGTCA |
| *Il1b* Fwd | CCCTGCAGCTGGAGAGTGTGGA |
| *Il1b* Rev | TGTGCTCTGCTTGTGAGGTGCTG |
| *Ptgs2* Fwd | GGCGCAGTTTATGTTGTCTGT |
| *Ptgs2* Rev | CAAGACAGATCATAAGCGAGGA |
| *Bdnf* Fwd | TCATACTTCGGTTGCATGAAGG |
| *Bdnf* Rev | GACCTCTCGAACCTGCCC |
| *Adamts5* Fwd | GGAGCGAGGCCATTTACAAC |
| *Adamt5* Rev | GCGTAGACAAGGTAGCCCACTTT |
| *Mmp3* Fwd | TTGTCCCGTTTCCATCTCTCTC |
| *Mmp3* Rev | TTGGTGATGTCTCAGGTTCCAG |
| *Adamts4 Fwd* | GAGGAGGAGATCGTGTTTCCAG |
| *Adamts4 Rev* | CAAACCCTCTACCTGCACCC |
| *Mmp12 Fwd* | GCTTACCCCAAGCTGATTTCC |
| *Mmp12 Rev* | ATGTTTTGGTGACACGACGGA |
| *Mmp13 Fwd* | CTTCTTCTTGTTGAGCTGGAACTC |
| *Mmp13 Rev* | CTCTGTGGACCTCACTGTAGACT |
| *Ngf Fwd* | TGATCGGCGTACAGGCAGA |
| *Ngf Rev* | GCTGAAGTTTAGTCCAGTGGG |
| *Vegf Fwd* | CACTGGACCCTGGCTTTACT |
| *Vegf Rev* | GCAGTAGCTTCGCTGGTAGA |
| Annealing Temperature: 60°C | |
