# Supplementary material for: Diet-induced obesity leads to behavioral indicators of pain preceding structural joint damage in wild-type mice
Source: Arthritis Res Ther. 2021 Mar 22;23:93. doi: 10.1186/s13075-021-02463-5 (PMC7983381; doi:10.1186/s13075-021-02463-5)
Supplement: Supplementary file 5 — Additional file 5: Supplementary Table 3. Multiplex analysis of cytokines, chemokines and growth factors in serum (Continued). [file 13075_2021_2463_MOESM5_ESM.docx]

|  | **Chow** | **HF** | **Western** |  |  | **Chow** | **HF** | **Western** |  |  | **Chow** | **HF** | **Western** |  |  |
| --- | --- | --- | --- | --- | --- | --- | --- | --- | --- | --- | --- | --- | --- | --- | --- |
|  | **n=6** | **n=6** | **n=6** |  |  | **n=5** | **n=6** | **n=6** |  |  | **n=5** | **n=6** | **n=6** |  |  |
| **Analyte** | **Mean (SD)** | **Mean (SD)** | **Mean (SD)** | **p-value** |  | **Mean (SD)** | **Mean (SD)** | **Mean (SD)** | **p-value** |  | **Mean (SD)** | **Mean (SD)** | **Mean (SD)** | **p-value** |  |
| Eotaxin | 2219 (3826) | 2657 (3647) | 3069 (3747) | 0.93 |  | **823.7 (223.3)** | **711.4 (342.9)** | **306.2 (316.9) *#** | **0.03*** |  | 777.5 (279.9) | 818.8 (169.6) | 722.2 (196.3) | 0.74 |  |
| G-CSF | 339.4 (308.8) | 435.6 (342.7) | 442.2 (416) | 0.86 |  | 333.9 (297) | 449.6 (348.3) | 195.3 (139.3) | 0.31 |  | 116.6 (95.7) | 140.7 (88.5) | 206.7 (226) | 0.60 |  |
| GM-CSF | 78.9 (101.8) | 58.6 (83.3) | 67.4 (132.6) | 0.98 |  | 79.4 (92.0) | 55.8 (78.9) | 61.8 (64.6) | 0.88 |  | 33.2  (44.4) | 21.1 (55.6) | 59.0 (102.8) | 0.66 |  |
| IFNy | 25.4 (36.6) | 52.0 (113.5) | 89.7 (192.0) | 0.70 |  | 74.1 (142.8) | 11.7 (22.0) | 16.6 (18.2) | 0.38 |  | 13.4 (18.0) | 57.4 (118.2) | 20.8 (41.4) | 0.58 |  |
| IL-1A | 389.8 (152.8) | 347.7 (90.7) | 248.5 (221.2) | 0.33 |  | **423.4 (253.8)** | **149 (79.4)** | **109.4**  **(47.5) *#** | **0.01**** |  | 329.6 (182) | 399.7 (153.6) | 514.6 (631.4) | 0.74 |  |
| IL-2 | 109.3 (167.2) | 159.3 (325.4) | 164.7 (334.9) | 0.93 |  | 111.8 (105.0) | 51.5 (75.6) | 51.3 (75.3) | 0.43 |  | 56.6 (92.5) | 19.6 (38.5) | 41.2 (57.0) | 0.60 |  |
| IL-3 | 7.9  (8.8) | 9.4 (10.7) | 13.9 (23.6) | 0.79 |  | 3.9  (4.2) | 5.9  (6.5) | 4.8  (7.1) | 0.87 |  | 0  (0) | 0.3  (0.6) | 1.3  (3.0) | 0.49 |  |
| IL-4 | 2.5  (3.0) | 8.2 (16.7) | 7.9  (7.8) | 0.59 |  | 2.9  (4.7) | 3.0  (3.3) | 3.5  (5.2) | 0.97 |  | 0.1  (0.1) | 0.1  (0.2) | 5.9 (11.0) | 0.25 |  |
| IL-5 | 19.4 (16.9) | 37.3 (44.9) | 23.4 (17.5) | 0.56 |  | 27.1 (33.2) | 33.7 (37.9) | 22.2 (14.3) | 0.80 |  | 6.5  (3.6) | 6.4  (5.9) | 15.1 (15.2) | 0.26 |  |
| IL-7 | 24.6 (27.0) | 30.2 (33.1) | 260.7 (600.9) | 0.43 |  | 97.6 (177.8) | 32.4 (48.9) | 5.0  (9.2) | 0.32 |  | 1.5  (2.2) | 5.1  (9.7) | 104.0 (241.2) | 0.41 |  |
| IL-9 | 143.7 (145.9) | 139.7 (151.3) | 378.0 (724.7) | 0.57 |  | 235.6 (318.7) | 48.5 (36.6) | 105.8 (139.1) | 0.29 |  | 243.6 (334.6) | 82.6 (88.5) | 128.9 (112.0) | 0.42 |  |
| IL-12  (p40) | 36.9 (48.8) | 18.3 (22.2) | 35.6 (43.9) | 0.68 |  | 10.4 (14.6) | 13.0 (15.1) | 6.5  (9.5) | 0.69 |  | 0 (0) | 122.9 (291.5) | 0  (0) | 0.40 |  |
| IL-12  (p70) | 40.4 (45.8) | 62.7 (108.2) | 241 (310.2) | 0.17 |  | 28.2 (30.6) | 70.8 (129.8) | 62.4 (88.3) | 0.75 |  | 0 (0) | 15.6 (38.2) | 281.2 (657.5) | 0.42 |  |
| IL-13 | 137.6 (141.2) | 133.5 (147.7) | 210.2 (383.2) | 0.84 |  | 231.7 (356.4) | 80.3 (74.3) | 87.0 (111) | 0.43 |  | 62.9 (65.5) | 55.9 (32.4) | 107.3 (146) | 0.62 |  |
| IL-15 | 324.0 (357.3) | 316.1 (357.3) | 239.3 (294.7) | 0.89 |  | 1231 (2454) | 269.5 (473.2) | 58.6 (69) | 0.34 |  | 3.0  (6.8) | 140.4 (203.1) | 983.7 (2191) | 0.42 |  |
| IL-17 | 8.7  (8.1) | 6.0  (9.4) | 6.3  (9.7) | 0.86 |  | 4.8  (3.5) | 4.9  (5.4) | 3.8  (5.3) | 0.92 |  | 3.0  (1.9) | 2.8  (3.4) | 11.9 (22.8) | 0.46 |  |
| LIF | 4.8  (5.2) | 5.5  (6.1) | 5.1  (5.7) | 0.97 |  | 4.0  (6.7) | 4.3  (5.3) | 0.3  (0.4) | 0.31 |  | 0  (0) | 1.8  (3.5) | 11.1  (25.3) | 0.44 |  |
| LIX | 6937 (4608) | 8908 (5462) | 6397 (3134) | 0.61 |  | 9955 (8707) | 5235 (5285) | 4320 (4376) | 0.31 |  | 9699 (1509) | 10211 (1959) | 7224 (4547) | 0.24 |  |
| M-CSF | 35.3 (38.7) | 25.8 (31.7) | 24.1 (27.3) | 0.82 |  | 19.1 (25.3) | 23.0 (23.7) | 15.5 (17.0) | 0.84 |  | 0.6  (1.1) | 28.4 (44.7) | 0.9  (0.8) | 0.16 |  |
| MIG | 474.0 (417.7) | 345.3 (353.3) | 345.7 (344.2) | 0.79 |  | 287.6 (193.5) | 368.3 (436.5) | 130.2 (82.1) | 0.37 |  | 85.2 (36.7) | 142.1 (110.6) | 114.3 (58.0) | 0.49 |  |
| MIP-1A | 249.6 (373) | 333 (539.3) | 587.6 (1032) | 0.69 |  | 417.8 (401) | 178.9 (207.3) | 191.2 (150.7) | 0.28 |  | 261.4 (302.7) | 109.2 (133.0) | 257.8 (146.2) | 0.58 |  |
| MIP-1B | 126.5 (161.5) | 104.7 (126.1) | 144.1 (204.2) | 0.92 |  | 217.2 (328.9) | 97.1 (124.6) | 123.7 (130.1) | 0.62 |  | 88.3 (116.3) | 100.7 (155.2) | 210.6 (286.9) | 0.55 |  |
| MIP-2 | 236.8 (113.5) | 195.8 (93.1) | 256.3 (123.7) | 0.64 |  | 127.4 (21.6) | 149.8 (62.6) | 111 (47.7) | 0.40 |  | 168.8 (21.4) | 198.9 (69.6) | 161.7 (15.0) | 0.33 |  |
| RANTES | 49.6 (44.4) | 76.7 (57.5) | 96.0 (73.9) | 0.42 |  | 53.6 (69.5) | 59.1 (57.2) | 58.9 (30.2) | 0.98 |  | 25.13 (22.17) | 36.77 (44.93) | 52.65 (46.62) | 0.54 |  |
| Values are displayed in pg/mL and analyzed by one-way ANOVA. * indicates significantly different from chow diet and # is significantly different from HF diet by Tukey’s post-hoc test. P<0.05 is significant | | | | | | | | | | | | | | | |
