# Supplementary material for: Diet-induced obesity leads to behavioral indicators of pain preceding structural joint damage in wild-type mice
Source: Arthritis Res Ther. 2021 Mar 22;23:93. doi: 10.1186/s13075-021-02463-5 (PMC7983381; doi:10.1186/s13075-021-02463-5)
Supplement: Supplementary file 6 — Additional file 6: Supplementary Table 4. Association between behavioral indicators of pain and histological joint damage. [file 13075_2021_2463_MOESM6_ESM.docx]

|  | **Bivariate (r)** | | |  | **Multivariate (β, r^2^)** | | | |
| --- | --- | --- | --- | --- | --- | --- | --- | --- |
| **Parameter** | **IVD degeneration** | **Knee OA** | **Synovitis** |  | **IVD degeneration (β)** | **Knee OA (β)** | **Synovitis**  **(β)** | **Whole model (r^2^)** |
| **Behavioral** |  |  |  |  |  |  |  |  |
| Von Frey | 0.002 | 0.206 | 0.233 |  | - | - | - | 0.099 |
| Grip Force | 0.015 | **0.254*** | 0.248 |  | -6.838 | **-0.373*** | -0.606 | **0.159*** |
| Acetone | 0.135 | 0.038 | 0.029 |  | - | - | - | 0.018 |
|  |  |  |  |  |  |  |  |  |
| **Tail suspension** |  |  |  |  |  |  |  |  |
| Rearing | 0.089 | 0.042 | 0.154 |  | - | - | - | 0.038 |
| Self-Support | 0.012 | 0.047 | 0.183 |  | - | - | - | 0.050 |
| Immobility | 0.063 | 0.061 | 0.224 |  | - | - | - | 0.075 |
| Stretch | 0.019 | 0.001 | 0.021 |  | - | - | - | 0.006 |
| **Open Field** |  |  |  |  |  |  |  |  |
| Distance travelled | 0.090 | 0.109 | **0.341***** |  | 781.536 | -17.419 | **-175.96**** | **0.132**** |
| Rest time | 0.140 | 0.025 | **0.269*** |  | - | - | - | 0.127 |
| Rearing | 0.106 | 0.015 | **0.304*** |  | - | - | - | 0.12 |
